# Transactions of the American Medical Association

**Published:** 1848-11

**Authors:** 


*Transactions of the American Medical Association*—We learn by the "Medical News" that this volume is at length published. We have not yet received a copy, but hope soon to be able to lay a digest of its contents before our readers.

